# Early prosthetic valve endocarditis presenting as acute right coronary artery occlusion 1 month after aortic valve replacement: a case report

**DOI:** 10.1186/s44215-026-00257-2

**Published:** 2026-05-07

**Authors:** Koki Yokawa, Kazunori Yoshida, Ko Ishimoto, Taku Nakagawa, Yosuke Tanaka, Tomonori Higuma, Yoshihiro Oshima, Hidefumi Obo, Hidetaka Wakiyama

**Affiliations:** Department of Cardiovascular Surgery, Kakogawa Central City Hospital, 439, Kakogawa-cho Honmachi, Kakogawa City, Hyogo 675-8611 Japan

**Keywords:** Prosthetic valve endocarditis, Coronary embolization, Acute coronary syndrome, Aortic root replacement, Nonocclusive mesenteric ischemia

## Abstract

**Background:**

Prosthetic valve endocarditis (PVE) is a life-threatening complication following valve replacement and is often challenging to diagnose in the early postoperative period. Coronary embolization is a rare manifestation of infective endocarditis, and PVE presenting as acute coronary syndrome is exceptionally uncommon.

**Case presentation:**

A 73-year-old man underwent surgical aortic valve replacement with a 21-mm bioprosthetic valve. His postoperative course was uneventful, and he was discharged without anticoagulation therapy. One month later, he developed exertional dyspnea, gastrointestinal symptoms, and intermittent chest pain, which progressed to cardiogenic shock with severe bradycardia. Emergent coronary angiography revealed acute occlusion of the right coronary artery, and percutaneous coronary intervention was performed. Intravascular ultrasound and contrast-enhanced computed tomography revealed a low-echoic, low-attenuation lesion at the right coronary ostium, initially interpreted as thrombotic material. Despite successful revascularization, profound circulatory instability persisted. Subsequent echocardiography revealed prosthetic valve dehiscence with an annular abscess, confirming early PVE. Emergent surgery included annular reconstruction with a bovine pericardial patch, aortic root replacement, removal of the coronary stent, and coronary artery bypass grafting. Intraoperative hemodynamics remained unstable, necessitating postoperative veno-arterial extracorporeal membrane oxygenation support. The patient ultimately succumbed to non-occlusive mesenteric ischemia on postoperative day 10.

**Conclusions:**

This case illustrates a rare and complex presentation of early PVE manifesting as acute right coronary artery occlusion. Coronary imaging alone may be insufficient to differentiate infected vegetation from thrombus. Early valve-focused echocardiographic evaluation is essential in patients with recent valve surgery presenting with acute coronary events.

## Background

Infective endocarditis (IE) remains a life-threatening condition despite advances in antimicrobial therapy and cardiac surgery. Systemic embolic events are common complications of IE and contribute substantially to patient morbidity and mortality. Most embolic events involve the cerebral or splenic circulation, whereas coronary artery embolism is rare, accounting for less than 1%–3% of embolic complications [1, 2]. When coronary embolism does occur, it often manifests as acute coronary syndrome (ACS), making timely differentiation from atherosclerotic plaque rupture or acute coronary thrombosis particularly challenging.

Prosthetic valve endocarditis (PVE), particularly in the early postoperative period, is associated with aggressive local infection, annular destruction, and poor outcomes. Early PVE can progress rapidly and may present with atypical manifestations before classical signs of infection become apparent. Coronary embolization from vegetations originating on infected prosthetic valves is particularly rare and has been reported only sporadically.

 [2, 3]. Herein, we present a case of early PVE manifesting as acute right coronary artery (RCA) occlusion caused by vegetation embolization, in which definitive diagnosis was confirmed only after surgical exploration.

## Case presentation

The patient had undergone aortic valve replacement using a 21-mm INSPIRIS RESILIA aortic bioprosthesis (Edwards Lifesciences, Irvine, CA, USA) one month prior to the current presentation. His postoperative course was uneventful. Subsequently, he was discharged on postoperative day 15. Postoperative anticoagulation with warfarin was not administered following bioprosthetic valve implantation. Routine outpatient laboratory testing after discharge showed no apparent signs of systemic infection.

Several days before readmission, the patient developed exertional dyspnea, anorexia, diarrhea, and intermittent chest pain lasting approximately 30 min, the origin of which was initially unclear. With progressive fatigue and worsening dyspnea, emergency medical services were contacted. Upon arrival at the emergency department, he was found to be bradycardic and in cardiogenic shock. A temporary pacemaker was inserted, and emergent coronary angiography revealed acute occlusion of the RCA. Percutaneous coronary intervention (PCI) was immediately performed. Although inflammatory markers were elevated on admission, these findings were nonspecific in the context of cardiogenic shock and multi-organ dysfunction and did not clearly indicate infection at the time of presentation.

**Fig. 1:**
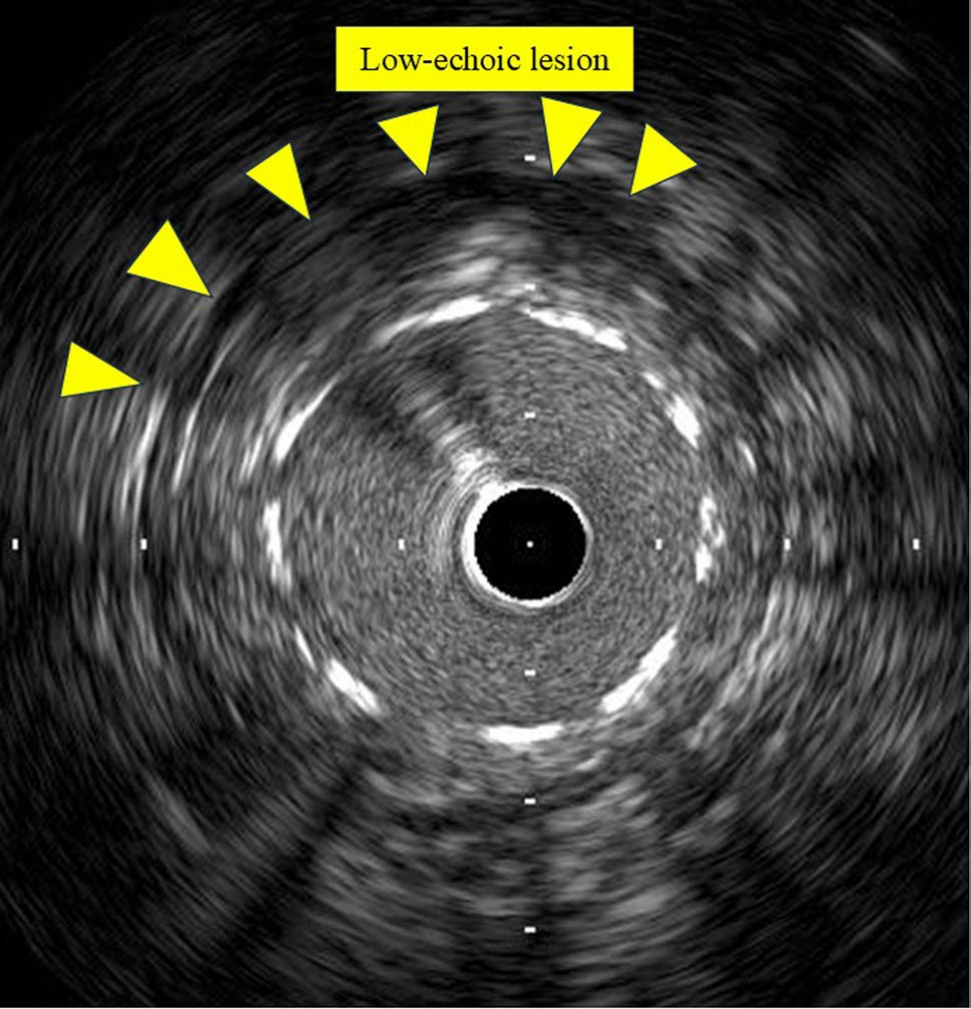
Intravascular ultrasound findings during percutaneous coronary intervention. Intravascular ultrasound obtained during percutaneous coronary intervention demonstrates a circumferential low-echoic lesion surrounding the deployed stent in the right coronary artery (yellow arrowheads). This finding was initially interpreted as thrombus; however, it was subsequently confirmed to represent infected vegetation. The imaging characteristics highlight the difficulty in distinguishing vegetation from thrombotic material in the acute setting

**Fig. 2:**
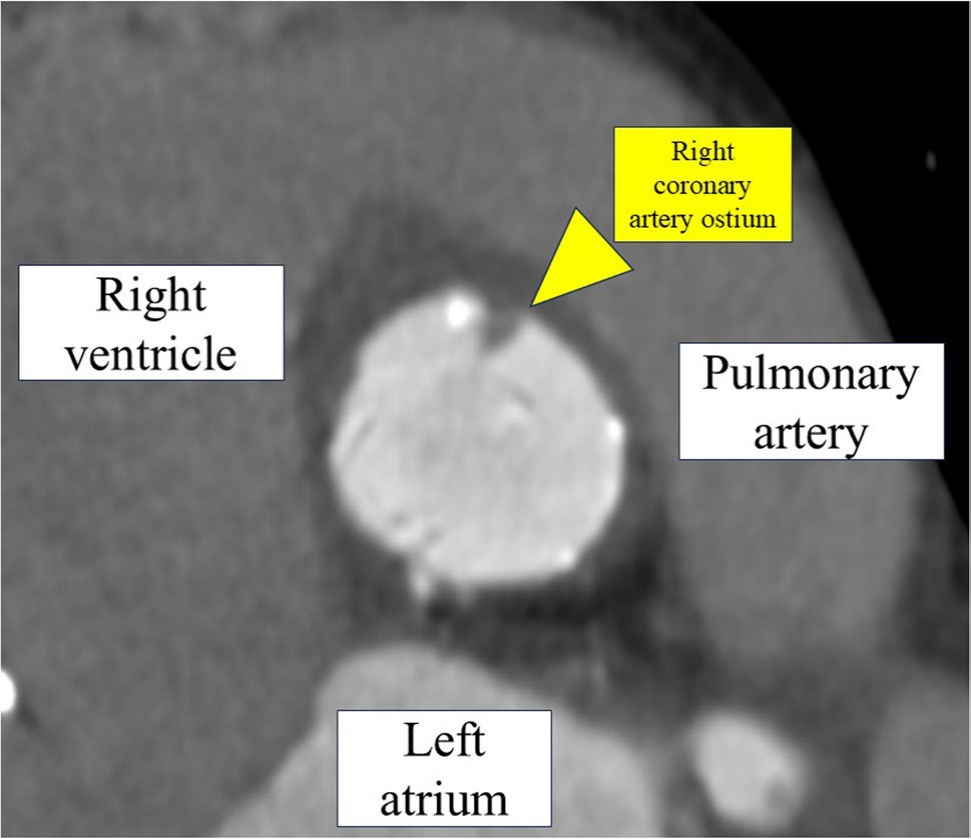
Contrast-enhanced computed tomography of the right coronary artery ostium. Contrast-enhanced computed tomography demonstrates a low-attenuation lesion at the ostium of the right coronary artery (yellow arrowhead). The anatomical relationships, including the right ventricle, pulmonary artery, and left atrium, are labeled for reference

Intravascular ultrasound obtained during the procedure revealed a low-echoic area surrounding the deployed stent in the RCA, initially interpreted as thrombotic material (Fig. 1). Contrast-enhanced computed tomography (CT) further revealed a low-attenuation lesion at the ostium of the RCA, apparently compressed by the implanted stent, findings also considered consistent with thrombus at that time (Fig. 2).

Despite successful coronary revascularization, the patient’s hemodynamic status remained profoundly unstable, accompanied by marked lactic acidosis. Abdominal ischemia was suspected, and exploratory laparotomy was performed shortly after PCI; however, no intestinal necrosis was detected. Veno-arterial extracorporeal membrane oxygenation and intra-aortic balloon pump support were initiated, and the patient was managed in the intensive care unit (ICU).

**Fig. 3:**
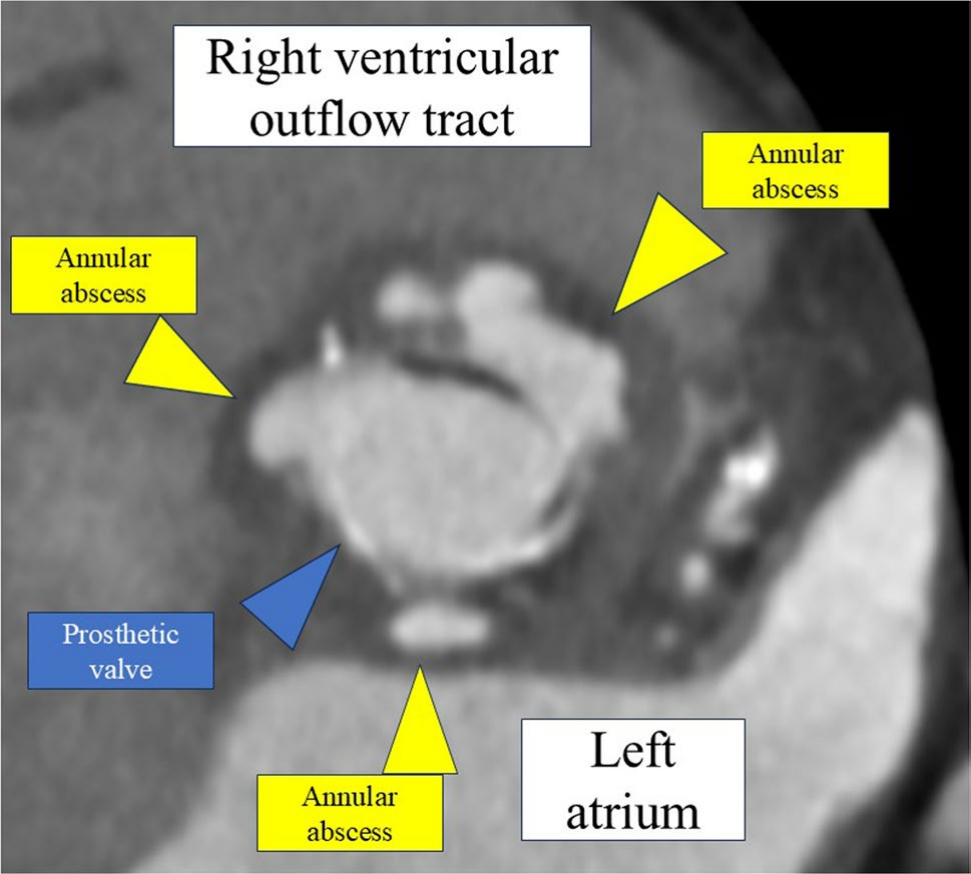
Contrast-enhanced computed tomography showing annular abscess formation. Contrast-enhanced computed tomography at the level of the aortic root reveals multiple low-attenuation lesions around the prosthetic valve consistent with annular abscess formation (yellow arrowheads). The prosthetic valve is indicated (blue arrowhead), and surrounding anatomical structures, including the right ventricular outflow tract and left atrium, are labeled

**Fig. 4:**
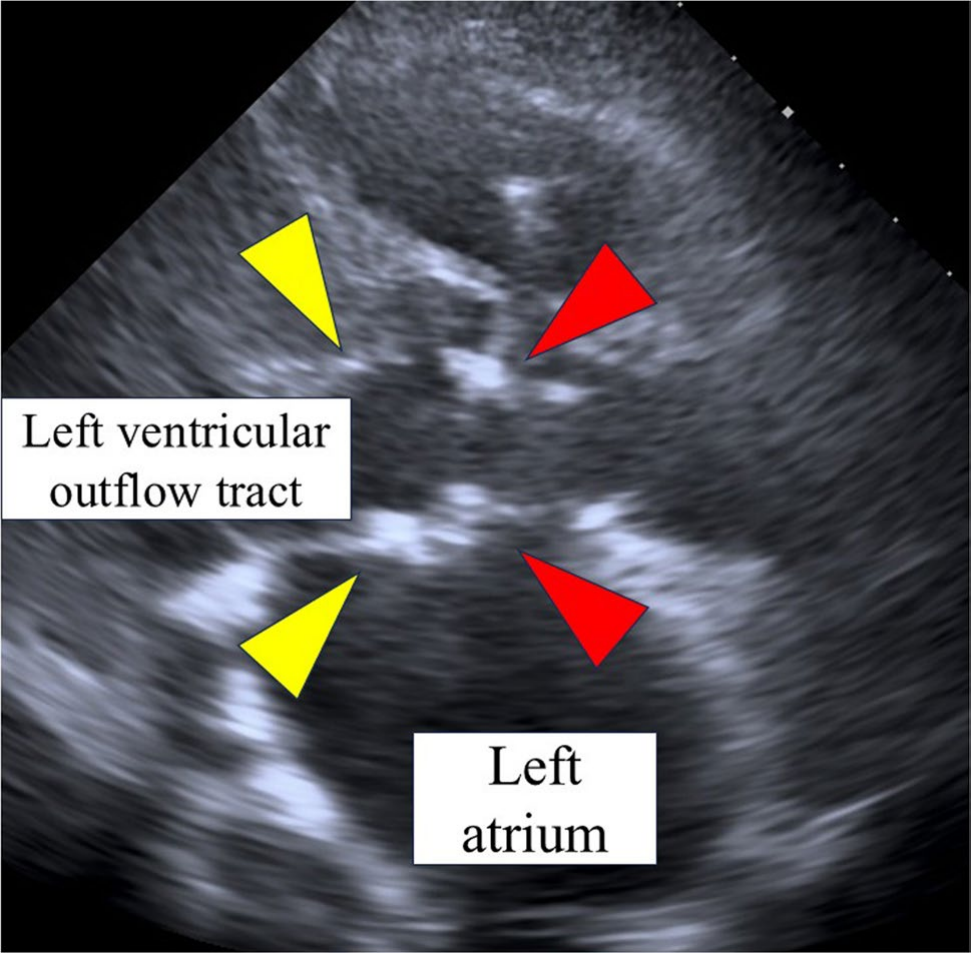
Transthoracic echocardiography before emergent surgery. Transthoracic echocardiography demonstrates the left ventricular outflow tract (yellow arrowheads) and the prosthetic aortic valve (red arrowheads). A clear separation between the prosthetic valve and the surrounding tissue is observed, indicating prosthetic valve dehiscence. These findings are highly suggestive of prosthetic valve endocarditis with annular involvement

Retrospective review of contrast-enhanced CT showed irregular low-attenuation areas around the aortic annulus on a vertical section through the left ventricular outflow tract, consistent with annular abscess formation, although these findings were not recognized during the initial evaluation (Fig. 3). Transthoracic echocardiography subsequently revealed prosthetic valve dehiscence with partial detachment of the prosthetic valve from the aortic annulus, strongly suggesting PVE (Fig. 4). Considering progressive valve instability and refractory shock, emergent surgical intervention was undertaken.

**Fig. 5:**
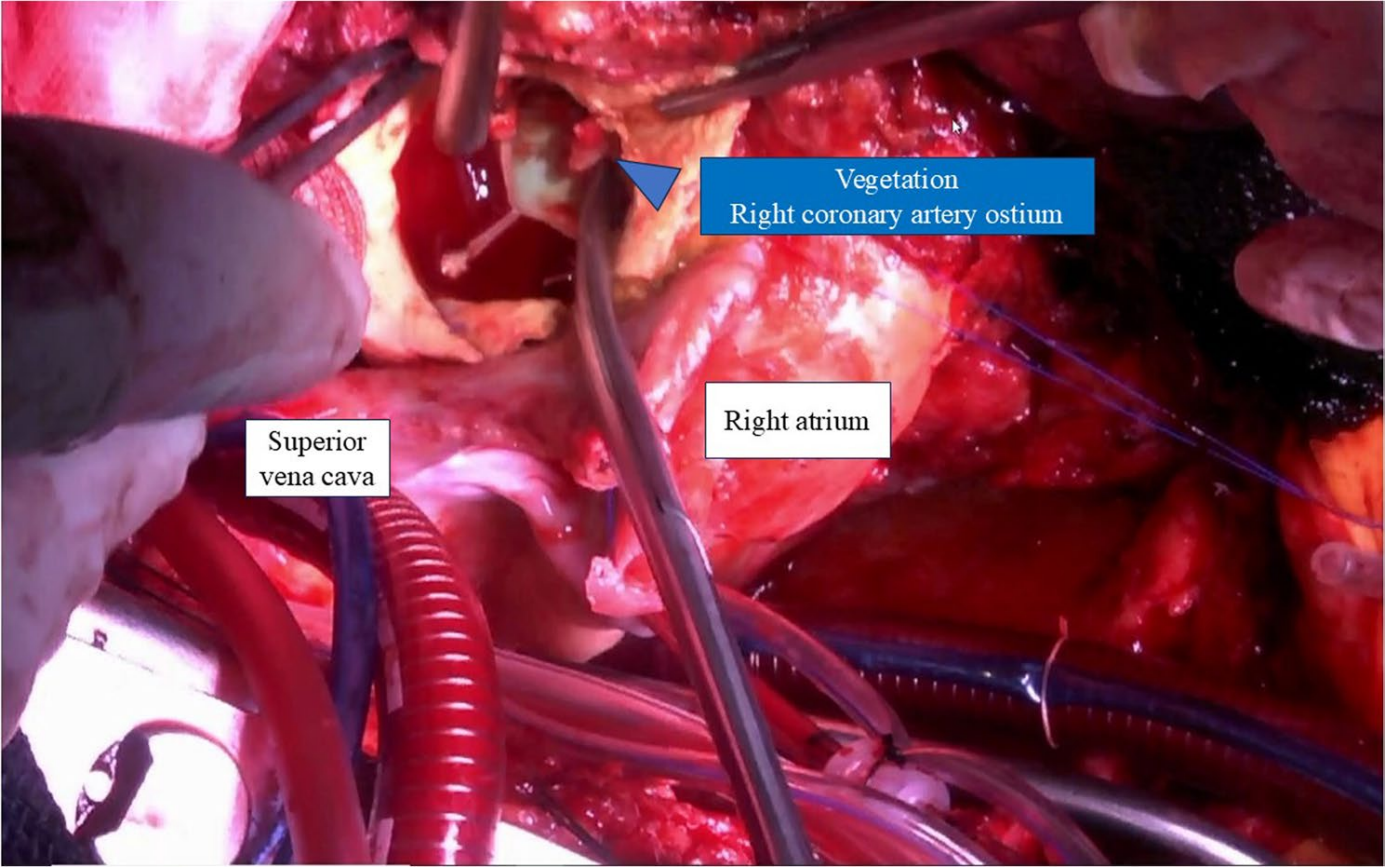
Intraoperative findings of vegetation impacted in the right coronary artery. Intraoperative photograph demonstrating infected vegetation impacted at the ostium of the right coronary artery (blue arrowhead). The superior vena cava and right atrium are labeled for anatomical orientation. Direct visualization of the vegetation confirms coronary embolization from prosthetic valve endocarditis as the mechanism of acute right coronary artery occlusion

Intraoperatively, near-circumferential destruction of the aortic annulus with extensive abscess formation was identified. The annulus was reconstructed using a bovine pericardial patch, and aortic root replacement was performed. The previously implanted RCA stent was removed due to concern for persistent infection, and coronary artery bypass grafting to the RCA was added. Infected vegetation impacting the ostium of the RCA was directly detected during surgery, confirming coronary embolization as the mechanism of the initial acute coronary occlusion (Fig. 5). Although histological examination of the embolic material was not performed, the retrieved material was submitted for microbiological analysis, which yielded methicillin-resistant coagulase-negative Staphylococcus (MRCNS). In addition, blood cultures obtained on admission were also positive for MRCNS.

Intraoperative hemodynamics remained unstable, and the patient returned to the ICU under continued VA-ECMO support. After successful weaning from extracorporeal membrane oxygenation on postoperative day 6, the patient initially showed transient hemodynamic stabilization. However, the patient subsequently developed progressive hypotension accompanied by abdominal distension and bloody output from the nasogastric tube, raising strong suspicion of intestinal ischemia. Contrast-enhanced computed tomography revealed no evidence of major mesenteric arterial occlusion; however, extensive ischemic changes were observed in the small intestine, gallbladder, and spleen. Laboratory findings demonstrated a rapid deterioration, including a marked increase in liver enzymes (AST/ALT: 44/59 to 704/316 U/L) and lactate dehydrogenase (LDH: 623 to 1144 U/L), along with a significant elevation in lactate levels (from 4.9 to 9.4 mmol/L), indicating severe systemic hypoperfusion. Based on these clinical, imaging, and laboratory findings, a diagnosis of non-occlusive mesenteric ischemia (NOMI) was made. Given the patient’s critically unstable hemodynamic condition, we determined that repeat exploratory laparotomy would not provide meaningful therapeutic benefit and would carry a prohibitive risk. Despite intensive supportive care, the patient’s condition progressively deteriorated, and the patient died on postoperative day 10.

### Discussion and conclusions

Systemic embolization is a well-known complication of IE; however, coronary artery embolism remains rare and is often underdiagnosed. Large cohort studies have shown that embolic events predominantly involve the cerebral and splenic circulations, whereas coronary embolization comprises only a small proportion of cases and is related to high mortality when it occurs [1].

Coronary embolism caused by septic vegetations presents a substantial diagnostic challenge because its clinical and angiographic features closely resemble those of acute coronary thrombosis [4]. Khan et al. presented a case of ST-elevation myocardial infarction secondary to IE in which differentiation between thrombus and vegetation was extremely challenging, even with contemporary imaging modalities [2]. This diagnostic ambiguity mirrors the present case, in which intravascular ultrasound and contrast-enhanced CT findings were initially interpreted as thrombus, making the initial diagnosis of acute coronary thrombosis reasonable.

ACS complicating IE has been reported only sporadically. In a recent review, Bouchlarhem et al. emphasized that ACS secondary to IE is rare but associated with poor outcomes, particularly when diagnosis and definitive infection control are delayed [3]. PCI may be unavoidable in patients presenting with hemodynamic collapse; however, PCI alone does not address the underlying infectious source and may complicate subsequent surgical management, particularly when coronary stents are implanted in an infected environment. Although histological confirmation of the embolus was not available, both the embolic material and blood cultures yielded MRCNS, strongly supporting the diagnosis of septic coronary embolization associated with prosthetic valve endocarditis rather than thrombotic occlusion. In this case, the absence of postoperative anticoagulation following bioprosthetic valve implantation may have contributed to the initial suspicion of prosthetic valve thrombosis. This factor complicated the differential diagnosis and may have delayed recognition of prosthetic valve endocarditis. This highlights the importance of appropriate postoperative anticoagulation and careful diagnostic assessment in similar clinical scenarios.

Furthermore, direct intraoperative visualization of vegetation impacted at the coronary ostium has been described only in exceptional cases. In this case, vegetation was directly observed at the RCA ostium during surgery, providing unequivocal confirmation that septic coronary embolization was the primary mechanism of the initial clinical presentation. The diagnosis of prosthetic valve endocarditis was particularly challenging in this case, as initial clinical and laboratory findings were nonspecific and could be attributed to cardiogenic shock and multi-organ failure. This underscores the importance of maintaining a high index of suspicion for infective endocarditis, even in the absence of definitive early signs of infection.

Management of PVE complicated by annular abscess requires radical debridement of infected tissue and secure reconstruction. Extensive annular destruction often necessitates aggressive surgical strategies beyond isolated redo valve replacement. In the present case, near-circumferential annular involvement precluded secure patch reconstruction alone, and aortic root replacement was required to achieve adequate infection control and structural stability.

Despite aggressive surgical and perioperative management, the patient ultimately died from nonocclusive mesenteric ischemia. This complication was considered a consequence of prolonged low-output state and severe circulatory failure rather than embolic phenomena. This case highlights the systemic severity of early PVE and underscores the limitations of even prompt and radical surgical intervention once profound circulatory collapse has occurred.

In conclusion, this case demonstrates that septic coronary embolism should be considered in the differential diagnosis of ACS occurring early after valve surgery. Differentiation between thrombus and vegetation may be extremely difficult using coronary imaging alone. Early valve-focused evaluation and multidisciplinary decision-making are essential to avoid delayed diagnosis in this rare but catastrophic clinical scenario.

## Data Availability

All data generated or analyzed during this study are included in this published article.

## Abbreviations

PVE: Prosthetic valve endocarditis
RCA: Right coronary artery
PCI: Percutaneous coronary intervention
ECMO: Extracorporeal membrane oxygenation
NOMI: Nonocclusive mesenteric ischemia
